# Supplementary material for: FtMYB163 Gene Encodes SG7 R2R3-MYB Transcription Factor from Tartary Buckwheat (Fagopyrum tataricum Gaertn.) to Promote Flavonol Accumulation in Transgenic Arabidopsis thaliana
Source: Plants (Basel). 2024 Sep 27;13(19):2704. doi: 10.3390/plants13192704 (PMC11478641; doi:10.3390/plants13192704)
Supplement: Supplementary file 1 [file plants-13-02704-s001.zip › Table S1.pdf]

Table S1. Basic information of *FtMYB163*.

Gene name	Locus tag	ORF length (bp)	Protein			Tm domains
			Length (No. of amino acids)	Mass (kDa)	pI	
FtMYB163	FtPinG0009153900.01	1119	286	42.18	6.23	0
