# Supplementary material for: FtMYB163 Gene Encodes SG7 R2R3-MYB Transcription Factor from Tartary Buckwheat (Fagopyrum tataricum Gaertn.) to Promote Flavonol Accumulation in Transgenic Arabidopsis thaliana
Source: Plants (Basel). 2024 Sep 27;13(19):2704. doi: 10.3390/plants13192704 (PMC11478641; doi:10.3390/plants13192704)
Supplement: Supplementary file 1 [file plants-13-02704-s001.zip › Table S2.pdf]

Table S2. The primers used in this study.

**Gene ID**	**Usage**	**Forward primer (5'-3')**	**Reverse primer (5'-3')**
FtPinG0009153900.01	*FtMYB163* for amplification	ATGGGAAGAGCACCTTGCTG	CTCAAGAAAGAAGCCAGGCAAC
FtPinG0009153900.01	*FtMYB163* for RT-PCR	GGGAGAGAGTAGCTGCAATG	CCCACATTAGCCACGATAGG
FtPinG0005405200.01	*FtActin7* for RT-PCR	ATGTTCACTACCACCGCTGA	TGAAC CTCTCAGCACCAATC
FtPinG0009153900.01	*FtMYB163* for overexpression	GGGGACAAGTTTGTACAAAAAAGCAGGC TCCATGGGAAGAGCACCTTGCTG	GGGGACCACTTTGTACAAGAAAGCTGGGTGCTCAAGAA AGAAGCCAGGCAA
FtPinG0009153900.01	*FtMYB163* for subcellular localization	CAAGCTTGCATGCCTGCAGGTCGACATGGGAAGAGCACC TTGCTG	CCTCGCCCTTGCTCACCATGGATCCAGAAAGAAGCCAGG CAACCA
FtPinG0009153900.01	*FtMYB163* for transgenic *Arabidopsis* PCR detection	ATCTCCACTGACGTAAGGGA	CTCAAGAAAGAAGCCAGGCAAC
FtPinG0009153900.01	*FtMYB163* for yeast hybridization	CCGGAAT TCATGGGAAGAGCACCTTGCTG	CGCGGATCCCTCAAGAAAGAAGCCAGGCAAC
At5g13930	*AtCHS* for RT-PCR	ACGGACATTTGAGGGAAGTTGG	AGGGTGGGCTATCCAGAAGAGG
At3g55120	*AtCHI* for RT-PCR	GCGGTTCTGGAATCTATCATCG	TCGTCCTTGTTCTTCATCATTAGC
At3g51240	*AtF3H* for RT-PCR	CTGACCCTGGAACCATTACCTT	CAGCATTCTTGAACCTCCCATT
At5g07990	*AtF3'H* for RT-PCR	ACCCGAGAGATTCTTACCCG	TCGAAATCGCTTCCTTTCACAT
At5g42800	*AtDFR* for RT-PCR	TGGTGGTCGGTCCATTCAT	GAGAGAGCGCGGTGATAAGG
At5g08640	*AtFLS* for RT-PCR	GCAATCCCGTTGGAGTTCATC	CTTCGTCGGGATCGCTTAGA
At4g22880	*AtANS* for RT-PCR	AGGTTAGGATTTCTTGGGCTGTG	CCGTGGAGGAAACTTAGCCG
At3g18780	*AtActin2* for RT-PCR	GGTATCGCTGACCGTATGAG	ATCTGCTGGAATGTGCTGAG
