# Supplementary figures and images for: FtMYB163 Gene Encodes SG7 R2R3-MYB Transcription Factor from Tartary Buckwheat (Fagopyrum tataricum Gaertn.) to Promote Flavonol Accumulation in Transgenic Arabidopsis thaliana
Source: Plants (Basel). 2024 Sep 27;13(19):2704. doi: 10.3390/plants13192704 (PMC11478641; doi:10.3390/plants13192704)

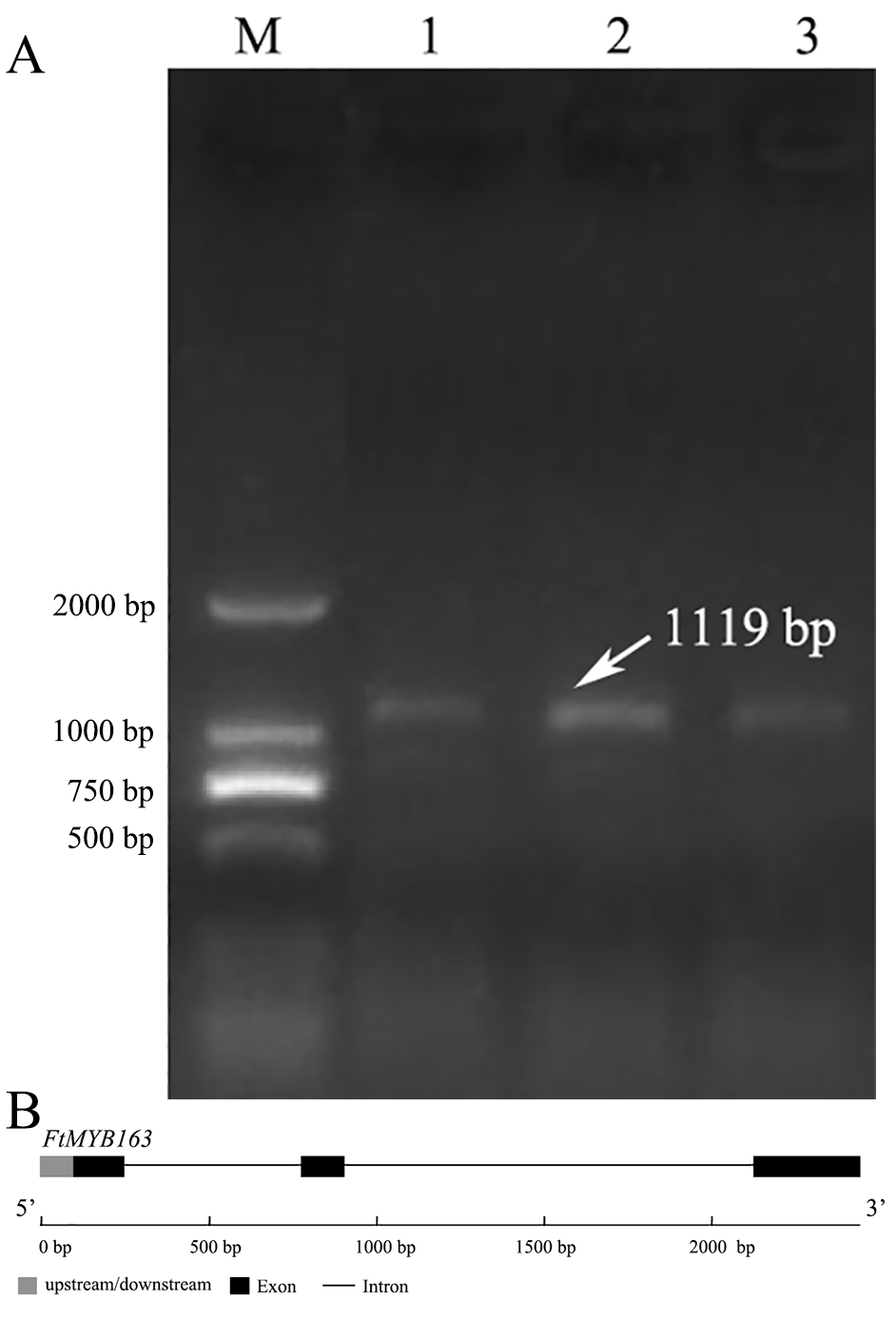

Supplement: Supplementary file 1 [file plants-13-02704-s001.zip › Fig. S1.tif]

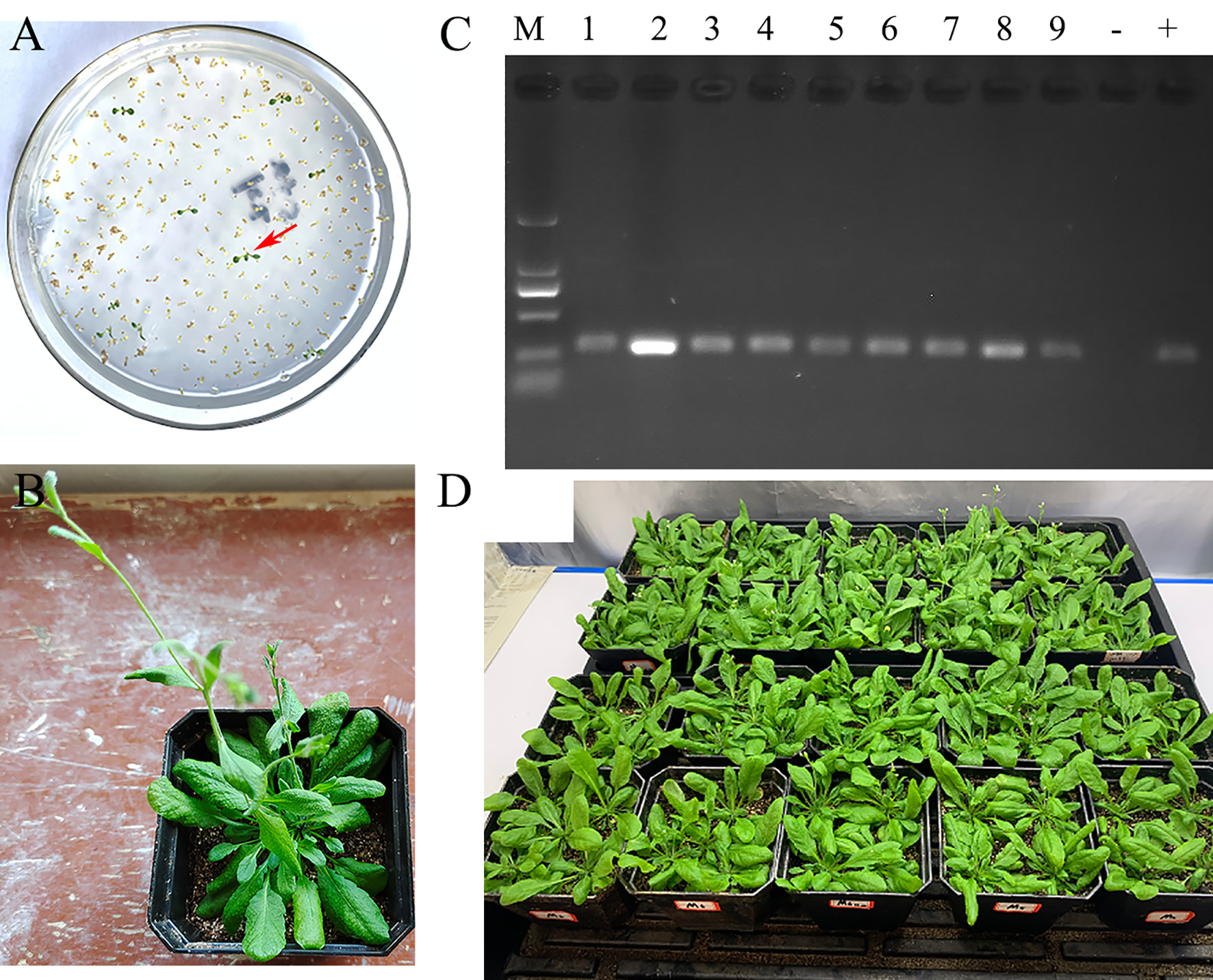

Supplement: Supplementary file 1 [file plants-13-02704-s001.zip › Fig. S2.tif]
